# The use of absolute values improves performance of estimation formulae: a retrospective cross sectional study

**DOI:** 10.1186/1471-2369-14-271

**Published:** 2013-12-05

**Authors:** Belén Redal-Baigorri, Knud Rasmussen, James Goya Heaf

**Affiliations:** 1 Department of Nephrology, Roskilde University Hospital, University of Copenhagen, Fjortenskæppevej 23, 4000, Roskilde, Denmark; 2 Department of Nephrology, Herlev University Hospital, University of Copenhagen, Herlev, Denmark

## Abstract

**Background:**

Estimation of Glomerular Filtration Rate (GFR) by equations such as Chronic Kidney Disease Epidemiology Collaboration (CKD-EPI) or Modification of Diet in Renal Disease (MDRD) is usually expressed as a Body Surface Area (BSA) indexed value (ml/min per 1.73 m^2^). This can have severe clinical consequences in patients with extreme body sizes, resulting in an underestimation in the case of obesity or an overestimation of GFR in the case of underweight patients. The aim of this study was to compare the performance of both estimation formula expressed in ml/min, instead of ml/min per 1.73 m^2^, with a reference method.

**Methods:**

Retrospective single centre cross sectional study of 185 patients. GFR was measured with ^51^Cr-EDTA and estimated with CKD-EPI and MDRD. Bias, precision and accuracy of absolute estimated GFR was calculated.

**Results:**

Bias of CKD-EPI and MDRD formulae expressed as an absolute value was 0.49 and 0.27 ml/min respectively, which is lower than previously reported. Precision was 12.95 and 16.33 and accuracy expressed as P30 was over 92.43% for CKD-EPI. There were no significant differences in GFR between the reference method and the estimation formulae.

**Conclusions:**

The performance of CKD-EPI and MDRD formulae can be significantly improved in the individual patient if the absolute values are used by removing the BSA normalization factor. Absolute estimated GFR by CKD-EPI is comparable to measured GFR, improving the performance of this formula in the assessment of individual kidney function, thus providing clinicians with an alternative to reference methods.

## Background

Glomerular filtration rate (GFR) is accepted as the best indicator of kidney function in clinical practice, and its measurement using urinary or plasma clearance of exogenous filtration markers is considered the gold standard [1]. In clinical practice, measuring GFR by these methods is impractical, expensive and in some cases unnecessary. This is one of the reasons why estimating equations have emerged.

Estimated GFR (e-GFR) is nearly always expressed as a Body Surface Area (BSA) indexed value (ml/min per 1.73 m^2^).

Measured GFR (m-GFR) can be expressed as an absolute value (ml/min) or a BSA indexed value (ml/min per 1.73 m^2^). These results are comparable as long as the patient has a BSA around 1.73 m^2^, which is the reference value in use over the last century. Whether this value of 1.73 m^2^ reflects a normal BSA nowadays, is disputable [2].

It is important for clinicians to be aware of the implications of using BSA indexed GFR values in populations with extreme BSA values, as suggested by an increasing amount of scientific papers investigating relationship between GFR and BSA over the last ten years [3-5].

Several studies have demonstrated that normalization of GFR with BSA in those cases with extreme body sizes might be misleading [6] and other studies have documented that obese patients have higher absolute GFR values than lean patients, independent of diabetes and hypertension [7-10].

There are different reasons why we keep using BSA as a normalization index of GFR, as already elegantly discussed by Delanaye [11], but the most important of them is to allow comparisons of different populations.

The impact of indexing GFR by BSA can be rather important if the results are to be used for drug dosing [12] or evaluation of kidney living donor in those patients outside the normal range of BSA [13]. In both cases, knowing the real individual kidney function is rather important. This is already recommended by the National Kidney Education Program (NKDEP) that suggests using the absolute GFR values in individual drug dosing of patients with extreme body sizes, as well as to consider measuring renal function with exogenous filtration markers when prescribing drugs with narrow therapeutic indices [14].

In practice very few are aware of these recommendations and most professionals use the BSA indexed GFR values.

For these reasons we decided to investigate whether the use of estimated absolute GFR values obtained with Modification of diet in renal disease (MDRD) and chronic kidney disease epidemiology collaboration (CKD-EPI) formula is comparable to absolute GFR values obtained with a gold standard method, thus avoiding professionals the need of investigating patients with expensive isotope methods, without compromising the quality of the results.

## Methods

Retrospective single centre cross-sectional study of patients who had their kidney function measured with a reference method, 51 chromium ethylene diamine tetraacetic acid clearance (^51^Cr-EDTA).

An analysis of differences between absolute GFR values obtained with ^51^Cr-EDTA and two creatinine-based estimation formulae: CKD-EPI and MDRD was performed.

The study was approved by the Danish Data Protection Agency (j.nr 2007-41-1006) and conducted in agreement with the principles of the Declaration of Helsinki.

### Participants

A total of 362 patients were referred to the local Department of Nuclear and Clinical Physiology in order to determine GFR by ^51^Cr-EDTA over a 2 year period. All patients were examined at the same laboratory.

The inclusion criteria were: Age ≥ 18; a minimum of two serum creatinine values with less than 15% variation in range within a period of three months and a serum creatinine value measured a maximum of three weeks before the ^51^Cr-EDTA measurement. We only used the first value of ^51^Cr-EDTA in those cases where this test was performed more than once.

Exclusion criteria: Only one creatinine value determination; acute renal insufficiency; diabetic ketoacidosis; increased values of protein (plasma (P) protein > 90 g/l), glucose (P glucose > 17 mmol/l), bilirubin (P bilirubin > 65 micromole/l) or uric acid (P uric acid > 0.45 mmol/l); renal replacement therapy; pregnancy; amputation; treatment with cephalosporin, cimetidine, methyldopa or trimethoprim.

The study population had not been diagnosed with end-stage renal disease (ESRD), as renal replacement therapy was an exclusion criterion. None of the patients were undergoing treatment with erythropoietin (EPO) or active D vitamin at the time of inclusion.

A total of 189 consecutive patients fulfilled the inclusion criteria.

Four were excluded due to a high serum level of uric acid [15], leaving 185 patients included for further analysis.

This patient population is now included in a larger meta-analysis [16].

### Estimated glomerular filtration rate

#### Analysis of serum creatinine

Serum creatinine was measured with the Jaffé method (Abbott Architect C systems TMc8000, reagent 7D64) which was calibrated with Isotope Dilution Mass Spectrometry (IDMS).

#### Calculation of estimated glomerular filtration rate

The MDRD and CKD-EPI formulas have previously been validated in patients with CKD stage 3 or lower, defined as GFR ≤ 59 ml/min per 1.73 m^2^. Validation was set at this level because higher values are associated with increased imprecision.

*CKD-EPI equation:* e ‒ GFR = 141 × min (Scr/k, 1)ª × max (Scr/k, 1)^‒ 1.209^ × 0.993^Age^ × 1.018 [if female] × 1.159 [if black].

Where Scr is serum creatinine, k is 0.7 for females and 0.9 for males, a is -0.329 for females and -0.411 for males, min indicates the minimum of Scr/k or 1, and max indicates the maximum of Scr/k or 1.

##### Modification of diet in renal disease formula (14) based on serum creatinine (S_c_), age, gender and race

$$\begin{array}{ll} e‒\mathrm{GFR}\left(\mathrm{ml},/,\min,\;,\mathrm{per},\;,1.73,m^{2}\right) & =175*{\left(S_{\mathrm{cr}}/88.4\right)}^{‒1.154}*{\mathrm{age}}^{‒0.203}\\ & \left(*,\left(0.742\;\mathrm{if}\;\mathrm{female}\right),*,\left(1.21\;\mathrm{if}\;\mathrm{black}\right)\right). \end{array}$$

Where S_cr_ is in micromoles/l; 88.4 is the molecular weight of S_cr_ and age is stated in years.

Body surface area (BSA) was calculated from the DuBois & DuBois formula with height in centimeters rather than meters, as BSA(m^2^) = 0.007184 × height (cm)^0.725^ × weight (kg)^0.425^.

We looked at absolute values and indexed values; therefore we used these formulae to convert one to another.

##### Conversion of BSA indexed GFR values to absolute GFR values

$$\begin{array}{ll} \mathrm{Absolute}\;\mathrm{GFR}= & \left(\mathrm{BSA}\;\mathrm{indexed}\;\mathrm{estimated}\;\mathrm{GFR}\times \mathrm{Patients}\;\mathrm{BSA}\right)\\ & /1.73m^{2}=\mathrm{ml}/\min. \end{array}$$

##### Conversion of absolute GFR values to BSA indexed GFR values

$$\begin{array}{ll} \mathrm{BSA}\;\mathrm{Indexed}\;\mathrm{GFR} & =\left(\mathrm{absolute}\;\mathrm{GFR}\times 1.73m^{2}\right)/\mathrm{Patients}\;\mathrm{BSA}\\ & =\mathrm{ml}/\min\;\mathrm{per}\;1.73m^{2}. \end{array}$$

### Measured Glomerular Filtration Rate (m-GFR)

GFR was determined from the total (renal and extra-renal) ^51^Cr-EDTA plasma clearance by a simplified single-injection technique with a single-plasma sample [17].

### Statistics

Data was analyzed with Excel and Statistica 99 version, by StatSoft inc. All GFR measurements and estimates follow a normal distribution.

#### Bias, precision and accuracy of e-GFR

Bias in this study is an expression of systemic error of estimated absolute GFR and was defined as the mean difference between estimated and measured absolute GFR.

Precision is an expression of the random variation or spread of estimated GFR around the measured GFR. It was expressed as the standard deviation (SD) of the bias. Large width equals low precision. Both precision and bias were expressed as ml/min.

Accuracy is affected by both bias and imprecision and was defined as the percentage of estimated GFR values within 10% and 30% limits of the measured GFR. Values over 90% are considered as indicators of high accuracy.

#### Comparison of absolute m-GFR versus absolute e-GFR

We investigated whether there were differences between measured and estimated GFR whenever results were expressed independently from BSA, in other words, GFR was expressed as an absolute value (ml/min) rather than as a BSA indexed value (ml/min per 1.73 m^2^).

Our hypothesis was that there were no differences in between the absolute m-GFR by ^51^Cr-EDTA or absolute e-GFR by CKD-EPI or MDRD.A T test for unpaired data was used, values of P <0.05 were considered significant.

We also performed an analysis where the BSA indexed values where compared against the absolute values for all the three methods to ascertain whether the bias found with ^51^Cr-EDTA, was also confirmed with CKD-EPI and MDRD formulae. We also analyzed data with a Bland Altman plot as this is the recommended analysis to compare methods.

## Results

In this study we looked at the performance of these two formulae whenever the results are expressed in absolute values, to avoid a possible influence of BSA in the results. In a previous similar study based on the same set of data we looked at similar results but only using the BSA indexed values [17].

### Patient characteristics

As shown in Table 1, the mean patient age exceeded 60 years, with a range of 26 to 83 years. There were more females than males. The mean absolute m-GFR was 88 ± 25 ml/min, (Range 33–156 ml/min) which means that there were no patients with CKD IV and V included in this study.

**Table 1:** Patient characteristics

**Patients (n)**	185
**Mean age ± SD, years**	63 ± 10
**Female, n (%)**	104 (56)
**Male, n (%)**	81 (44)
**Mean creatinine, mmol/L ± SD**	73 ± 21
**Mean m-GFR ± SD ml/min**	88 ± 25
**Mean e-GFR by CKD-EPI ± SD ml/min**	89 ± 21
**Mean e-GFR by MDRD ± SD ml/min**	88 ± 25
**Mean BMI, kg/m**^**2**^	24.2
**Mean BSA ± SD, m**^**2**^	1.78 ± 0.20

*SD*: Standard deviation; *m-GFR*: Measured glomerular filtration rate; *e-GFR*: Estimated glomerular filtration rate; *CKD-EPI*: Chronic kidney disease epidemiology collaboration formula; *MDRD*: Modification of diet in renal disease formula. *BMI*: Body mass index; *BSA*: Body surface area.

32 (17%) patients had m-GFR > 110 ml/min, with a median of 120 ml/min (111–156).

There were 23 (12%) patients with a GFR less than 60 ml/min (Range 33–59), median 49 ml/min.

The average BSA in this study was 1.78 ± 0.20 m^2^, higher than the standard BSA of 1.73 m^2^, with a range of 1.23 to 2.30 m^2^. In comparison BMI mean was 24.2 kg/m^2^ and range 15.6 to 36.4 kg/m^2^.

### Bias, precision and accuracy results of e-GFR with CKD-EPI and MDRD formulae

With regards to CKD-EPI, Table 2 shows that bias was lower than in the previous study based on BSA indexed data (0.49 vs. 1.16), precision was also slightly better (12.95 versus 13.37) but the best results arose from the higher P30 which crossed the level of 90%, considered as the reference value for a good accuracy (92.43% versus 89.73%) [18].

**Table 2:** Bias, precision and accuracy results of e-GFR with CKD-EPI and MDRD

			**n**	**Bias**	**Precision**	**IQR**	**P10**	**P30**
***Whole group***	**MDRD**	**ml/min**	185	0.27 (-2.09 to 2.64)	16.33	20.27	52.43%	88.11%
	**CKD-EPI**	**ml/min**	185	0.49 (-1.38 to 2.37)	12.95	17.28	52.43%	92.43%
***BSA < 1.60 m***^***2***^	**MDRD**	**ml/min**	34	3.18 (-1.76 to 8.14)	14.20	17.67	47.06%	82.35%
	**CKD-EPI**	**ml/min**	34	3.66 (-0.82 to 8.14)	12.85	18.81	47.06%	79.41%
***BSA < 1.80 m***^***2***^	**MDRD**	**ml/min**	81	-1.64 (-4.58 to 1.30)	13.29	17.32	56.79%	97.53%
	**CKD-EPI**	**ml/min**	81	-0.68 (-3.30 to 1.94)	11.85	14.67	56.79%	98.77%

*CKD-EPI*: Chronic Kidney Disease Epidemiology Collaboration formula; *MDRD*: Modification of diet in renal disease formula; *BSA*: Body surface area. *IQR*: interquartile range; *P10 and P30*: accuracy within 10 and 30% limits of the measured glomerular filtration rate; *BSA*: body surface area.

The bias for the whole group was even lower for MDRD, but the P30 was not above 90% and precision was lower than for CKD-EPI.

When looking at the same analysis stratified by different levels of BSA, we found that in the group of patients with BSA more than 1.80 m^2^ with CKD-EPI formula, there is an improvement in the results as compared to MDRD, with a bias of -0.68 ml/min, a high precision (11.85 vs. 12.95) and a higher P10 (56.79 vs. 52.43%) as well as P30 (98.77 vs. 92.43%).

### Comparison of measured absolute values versus estimated absolute values

Table 3 shows the bias between absolute values obtained with the gold standard method and both estimation formulae. In other words, the normalization factor was removed.

**Table 3:** Comparison of CKD-EPI and MDRD versus 51Cr-EDTA as absolute GFR values (ml/min)

	**All participants**				**Women**				**Men**			
	**N**	**Mean bias (95% C.I)**	**Std dev.**	**P value**	**N**	**Mean bias (95% C.I)**	**Std dev.**	**P value**	**N**	**Mean bias (95% C.I)**	**Std dev.**	**P value**
**CKD-EPI versus 51-CR-EDTA ml/min**												
all	185	0.49 (-1.38 to 2.37)	12.95	0.83	104	0.86 (-1.70 to 3.44)	13.23	0.73	81	0.01 (-2.78 to 2.81)	12.65	0.99
BSA < 1.60	34	3.66 (-0.82 to 8.14)	12.85	0.35	32	2.65 (-1.87 to 7.18)	12.56	0.52	2	19.76 (-0.03 to 39.57)	2.2	<0.05
BSA 1.60-1.79	70	0.31 (-3.05 to 3.67)	14.11	0.91	52	-0.70 (-4.69 to 3.27)	14.31	0.81	18	3.25 (-3.42 to 9.94)	13.44	0.66
BSA > 1.80	81	-0.68 (-3.30 to 1.94)	11.85	0.86	20	2.10 (-3.18 to 7.40)	11.31	0.74	61	-1.59 (-4.65 to 1.47)	11.97	0.73
**MDRD versus 51CR-EDTA ml/min**												
all	185	0.27 (-2.09 to 2.64)	16.33	0.91	104	-1.66 (-4.70 to 1.38)	15.66	0.53	81	2.77 (-0.97 to 6.51)	16.93	0.53
BSA < 1.60	34	3.18 (-1.76 to -8.14)	14.20	0.46	32	2.24 (-2.83 to 7.33)	14.10	0.62	2	18.25 (-3.65 to 40.16)	2.43	<0.05
BSA 1.60-1.79	70	1.08 (-3.69 to 5.86)	20.02	0.76	52	-3.13 (-7.88 to 1.62)	17.08	0.35	18	13.26 (1.69 to 24.83)	23.27	0.17
BSA > 1.80	81	-1.63 (-4.58 to 1.30)	13.29	0.69	20	-4.09 (-10.45 to 2.25)	13.57	0.52	61	-0.83 (-4.21 to 2.55)	13.22	0.86

*CKD-EPI*: Chronic Kidney Disease Epidemiology Collaboration formula; *MDRD*: Modification of diet in renal disease formula; *BSA*: Body surface area.

This analysis showed that there were no differences in between GFR values obtained with ^51^Cr-EDTA and CKD-EPI or MDRD estimation formulae, as long as the absolute values are used.

An analysis of data stratified by BSA showed that there was a clinical significant difference in the group of males with BSA < 1.60 m^2^ for both formula, but there were only 2 participants in the group. This difference was disregarded.

**Figure 1:**
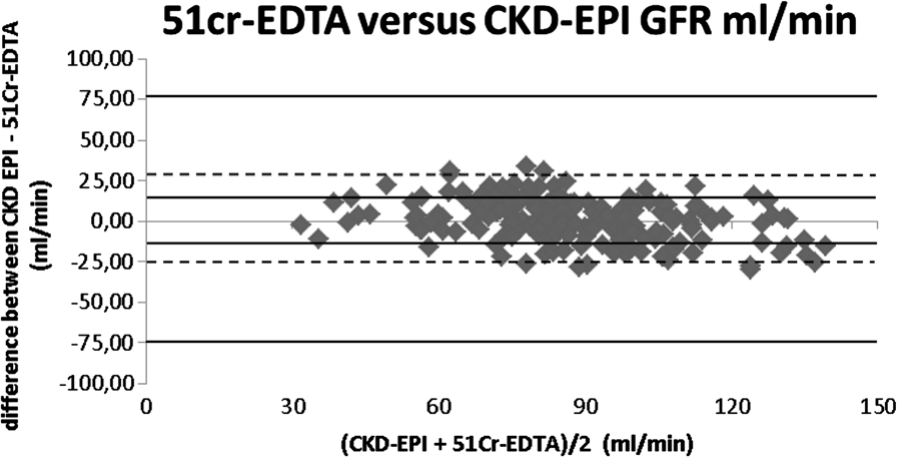
**Bland altman plot 51cr-EDTA versus CKD-EPI formula.** 51cr-EDTA: 51 chromium ethylene diamine tretraacetic acid clearance; CKD-EPI: Chronic kidney disease epidemiology collaboration.

**Figure 2:**
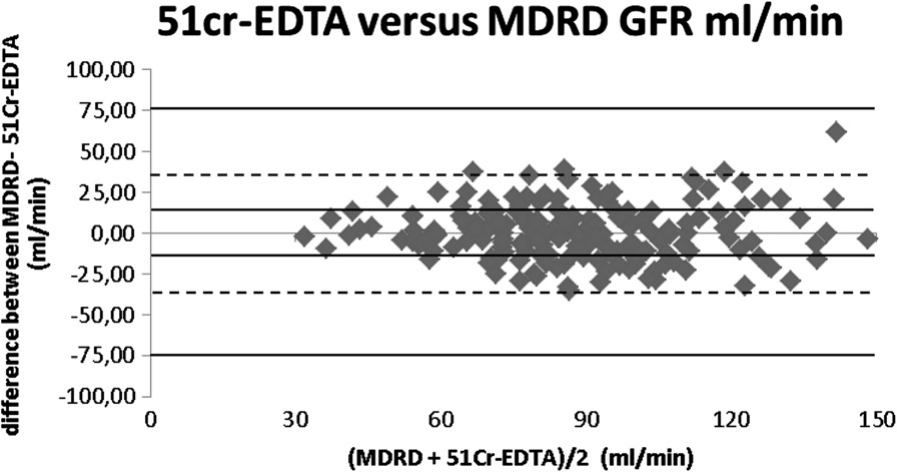
**Bland altman plot 51cr-EDTA versus MDRD formula.** 51cr-EDTA: 51 chromium ethylene diamine tretraacetic acid clearance; MDRD: Modification of diet in renal disease.

By looking at the data in a graphic manner with Bland-Altman plot it is quite clear to see that the comparison between ^51^Cr-EDTA and CKD-EPI (Figure 1), demonstrates that most values are around 0 with a lesser spreading of data and no outliners. Data for MDRD is more spread, showing that this method is less comparable to ^51^Cr-EDTA (Figure 2).

### Comparison of BSA indexed and absolute GFR values for all three methods

When differences in between indexed and absolute values for all three methods were analyzed, as shown in Table 4, a very similar bias was found across all three methods, suggesting that the bias is related to the BSA normalization factor.

**Table 4:** **Differences in between BSA indexed GFR (ml/min per 1.73 m**^**2**^**) and absolute GFR values (ml/min) for 51Cr-EDTA, CKD-EPI and MDRD**

	**All**				**Women**				**Men**			
	**n**	**Mean bias (95% C.I)**	**Std dev.**	**P value**	**n**	**Mean bias (95% C.I)**	**Std dev.**	**P value**	**n**	**Mean bias (95% C.I)**	**Std dev.**	**P value**
**51cr-EDTA**												
**all**	185	-3.01 (-4.57 to -1.46)	11	<0.001	104	2.42 (0.90 to 3.94)	7.81	<0.01	81	-10.09 (-12.18 to -7.83)	9.82	<0.001
**BSA < 1.60**	34	10.23 (8.57 to 11.90)	4.76	<0.001	32	10.48 (8.74 to 12.21)	4.80	<0.001	2	6.37 (-0.06 to 12.76)	0.71	0.0505
**BSA 1.60-1.79**	70	1.55 (0.84 to 2.25)	2.95	<0.001	52	1.84 (1.05 to 2.63)	2.83	<0.001	18	0.70 (-0.89 to 2.29)	3.2	0.37
**BSA > 1.80**	81	-12.53 (-14.26 to -10.79)	7.84	<0.001	20	-8.95 (-11.28 to -6.63)	4.97	<0.001	61	-13.70 (-15.82 to -11.58)	8.28	<0.001
**CKD-EPI**												
**all**	185	-2.75 ( -4.31 to -1.19)	10.75	<0.001	104	2.67 (1.07 to 4.27)	8.22	<0.01	81	-9.73 (-11.84 to -7.61)	9.57	<0.001
**BSA < 1.60**	34	11.08 (9–05 to 13.11)	5.82	<0.001	32	11.24 (9.09 to 13.39)	5.96	<0.001	2	8.51 (-1.30 to 18.33)	1.09	0.057
**BSA 1.60-1.79**	70	1.57 (0.86 to 2.28)	2.97	<0.001	52	1.87 (1.09 to 2.64)	2.78	<0.001	18	0.71 (-0.98 to 2.40)	3.4	0.38
**BSA > 1.80**	81	-12.31 ( -13.94 to -10.68	7.37	<0.001	20	-8.94 (-11.02 to -6.86)	4.44	<0.001	61	-13.41 ( -15.41 to -11.41)	7.82	<0.001
**MDRD**												
**all**	185	-2.68 ( -4.26 to -1.11)	10.85	<0.001	104	2.80 (1.21 to 4.38)	8.15	<0.001	81	- 9.72 (-11.89 to -7.55)	9.5	<0.001
**BSA < 1.60**	34	11.10 (8.87 to 13.32)	6.38	<0.001	32	11.27 (8.91 to 13.63)	6.54	<0.001	2	8.53 (-1.60 to 18.30)	1.1	0.059
**BSA 1.60-1.79**	70	1.63 (0.87 to 2.39)	3.20	<0.001	52	1.85 (1.07 to 2.63)	2.80	<0.001	18	0.98 (-1.08 to 3.06)	4.16	0.32
**BSA > 1.80**	81	-12.20 ( -13.86 to -10.54)	7.52	<0.001	20	-8.30 (-10.19 to -6.41)	4.04	<0.001	61	-11.44 (-15.52 to -12.50)	7.97	<0.001

*51Cr-EDTA*: 51 chromium ethylene diamine tetraacetic acid clearance; *CKD-EPI*: Chronic Kidney Disease Epidemiology Collaboration formula; *MDRD*: Modification of diet in renal disease formula; *BSA*: Body surface area.

There was a significant bias in all three groups as a whole as well as whenever BSA was > 1.80 m^2^.

These results showed that in patients with the lowest BSA under 1.60 m^2^ there was a significant overestimation of GFR patent with all three methods, demonstrating that the tendency can be found with the gold standard method as well as the estimation formula.

There were no clinical or statistical differences between mean bias for patients with at BSA <1.60 m^2^ measured with ^51^Cr-EDTA (10.23 ± 4.76 ml/min) and mean bias for the same group calculated with CKD-EPI (11.08 ± 5.82 ml/min) as p value was 0.51.

The same results applied to the comparison between ^51^Cr-EDTA and MDRD where p value was 0.53.

The opposite effect was observed in the group of patients with BSA over 1.80 m^2^, as there was an underestimation of the measured GFR with all three methods if the BSA indexed GFR value was used.

Again there were no differences in the mean bias between all three methods, as underestimation of GFR by ^51^Cr-EDTA of -12.53 ± 7.84 ml/min compared to underestimation by CKD-EPI of -12.31 ± 7.37 ml/min (n = 81) showed a p value of 0.85. Same analysis with MDRD values showed a p value of 0.78.

Table 5 shows the analysis of bias between ^51^Cr-EDTA and the two estimation methods stratified by age. The chosen range is 25 to >70 years, as the youngest patient was 25.64 and the oldest was 83.42 years old. As demonstrated in Table 3 by BSA stratification, no differences are found in the 3 chosen groups. The best results can be observed with CKD-EPI formula in the group of patients with an age range between 51 and 70 years, as bias is as low as 0.07 and no significant clinical or statistical differences between the reference method and the estimation formula are found.

**Table 5:** Comparison of CKD-EPI and MDRD versus 51Cr-EDTA as absolute GFR values (ml/min) stratified by age

	**All participants**				
	**n**	**BSA**	**Mean bias (95% C.I)**	**Std dev.**	**P value**
	**CDK-EPI VERSUS 51-CR-EDTA ml/min**				
**All**	185	1.78 ± 0.20	0.49 (-1.38 to 2.37)	12.95	0.83
**25-50 years**	22	1.88 ± 0.20	-1.18 (-7.22 to 4.86)	13.63	0.87
**51-70 years**	120	1.77 ± 0.20	0.07 (-2.45 to 2.59)	13.97	0.98
**> 70 years**	43	1.75 ± 0.20	2.55 (-0.25 to 5.34)	9.10	0.50
	**MDRD VERSUS 51CR-EDTA ml/min**				
**All**	185	1.78 ± 0.20	0.27 (-2.09 to 2.64)	16.33	0.91
**25-50 years**	22	1.88 ± 0.20	3.18 (-1.76 to -8.14)	14.20	0.46
**51-70 years**	120	1.77 ± 0.20	1.08 (-3.69 to 5.86)	20.02	0.76
**> 70 years**	43	1.75 ± 0.20	-1.63 (-4.58 to 1.30)	13.29	0.69

CKD-EPI: Chronic Kidney Disease Epidemiology Collaboration formula; MDRD: Modification of diet in renal disease formula; BSA: Body surface area.

## Discussion

To our knowledge this is the first clinical study of a group of patients in a creatinine steady state, comparing the performance of MDRD and CKD-EPI GFR estimation equation expressed in absolute values and not BSA indexed values, with a reference method for measuring GFR.

The absolute GFR values obtained with both estimation formulae are very similar to the results obtained with ^51^Cr-EDTA, suggesting that the performance of both formulae can be improved if the BSA normalization factor is removed, but in general CKD-EPI performance was slightly better measured with different parameters.

This study also shows what other studies have suggested, with increasing BSA there is an underestimation of GFR as long as the BSA indexed values are used, what is new in this study is that this underestimation is eliminated if the absolute values are used, improving the performance of CKD-EPI and MDRD formulae in all groups regardless BSA.

The best results regarding bias, precision and accuracy were obtained for CKD-EPI formula in the group of patients with BSA >1.80, showing a P30 of 98.77%, and an underestimation of only 0.68 ml/min with regards to ^51^Cr-EDTA.

By looking at the different age groups, the best results were also found with the CKD-EPI formula in the range of 51 to 70 years with a bias as low as 0.07 ml/min.

The Bland Altman plots show that the best results are seen with CKD-EPI, with a good concentration of values around the bias line.

A review about BSA indexing renal function parameters by Delanaye et al. [19] has argued against using BSA indexed measured GFR values, especially if the results are to be used for drug dosing in an obese population or GFR longitudinal follow-up purposes, but they do not recommend to “de-index” MDRD in patients with a BSA over 2.4 m^2^, because the MDRD formula was based on patients with a BSA range between 1.5 and 2.4 m^2^.

In this study we demonstrate that de-indexation of estimated GFR by MDRD or CKD-EPI follows the same trend as absolute measured GFR by ^51^Cr-EDTA, but our population has a BSA range of 1.23 to 2.30 m^2^.

By expressing the raw estimated GFR values, factors such as cachexia or obesity are removed, and that might avoid under and overestimation largely observed in many studies, which could contribute to a more accurate dose calculation of GFR based drugs, such as carboplatin and a reduction of the inaccuracies in drug dosing reported by other studies [5,20].

There are many studies looking at the problems related to BSA based GFR estimation formula, suggesting other parameters such as lean body mass [3] but if the goal of estimating GFR is drug dosing or an individual assessment of kidney function, the absolute values without further correction might be more accurate and easy to calculate.

In those cases where GFR is used to compare populations, the use of extracellular volume (ECV) as normalization factor seems to be more adequate as BSA, body mass index (BMI) and lean body mass are all related to weight and height, but measuring ECV is not feasible in daily practice [4].

## Conclusions

In view of these facts we recommend clinicians to convert the estimated GFR values obtained with CKD-EPI to the absolute GFR values whenever the individual kidney function needs to be investigated, as bias with regards to the reference method is rather low. These considerations might also apply to other groups of patients when calculating dose of nephrotoxic drugs, as recommended by NKDEP or in assessing GFR for other purposes such as suitability for living kidney donation [13] or indication for renal replacement therapy.

Relevant organizations might consider expressing GFR estimation equations as absolute values as well as BSA indexed values, leaving clinicians the opportunity to use the most relevant value for a given patient in different clinical situations [21].

A prospective study looking at the impact in chemotherapy drug dosing intention by using GFR estimation formula expressed in absolute values is desirable, as such an study might show whether there are clinical consequences or not.

## Competing interests

We declare that that the results presented in this paper have not been published previously in whole or part, except in abstract format.

## Authors’ contributions

BR is responsible for conception and design, data acquisition, analysis and interpretation, drafting and revision as well as final approval of the manuscript. KR is responsible for conception and design, manuscript drafting and revision as well as final approval of the manuscript. JH is responsible for drafting and revising the manuscript, analysis and interpretation of data as well as final approval of the manuscript. All authors read and approved the final manuscript.

## Pre-publication history

The pre-publication history for this paper can be accessed here:

http://www.biomedcentral.com/1471-2369/14/271/prepub
